# Heart Rate Variability and Heart Failure with Reduced Ejection Fraction: A Systematic Review of Literature

**DOI:** 10.2174/011573403X327105241021180916

**Published:** 2024-11-01

**Authors:** Michiaki Nagai, Hallum Ewbank, Yukiko Nakano, Benjamin J. Scherlag, Sunny S. Po, Tarun W. Dasari

**Affiliations:** 1 Cardiovascular Section, Department of Medicine, University of Oklahoma Health Science Center, Oklahoma, OK 73104, USA;; 2 Department of Cardiology, Hiroshima City Asa Hospital, Hiroshima, Japan;; 3 College of Medicine, University of Oklahoma Health Sciences Center, Oklahoma City, Oklahoma, OK 73104, USA;; 4 Department of Cardiovascular Medicine, Graduate School of Biomedical and Health Sciences, Hiroshima University, Hiroshima, Japan

**Keywords:** Heart rate variability, autonomic nervous, heart failure with reduced ejection fraction, sympathetic nervous system, vagus nervous system, HF phenotype

## Abstract

**Introduction:**

Autonomic impairment is a hallmark of heart failure with reduced ejection fraction (HFrEF). While there have been studies on general values for each index of heart rate variability (HRV) analysis in HFrEF, a systematic review comprehensively examining representative values in HFrEF is lacking.

**Methods:**

We searched PubMed, Embase, and Cochrane databases to extract studies reporting representative values of HRV metrics in HFrEF.

**Results:**

A total of 470 HFrEF patients from 6 studies were included in the review. In general, time and frequency domains were abnormally lower in HFrEF, portending a worse prognosis. In HFrEF, the mean or median value of the standard deviation of NN interval, root mean square successive difference, pNN50, and low-frequency power/high-frequency power were 40 to 121 msec, 19 to 62 msec, 1.3 to 14%, and 1.00 to 1.73, respectively.

**Conclusion:**

In this systematic review, most HRV metrics were found to be calculated from 24-hour Holter recordings and were lower in HFrEF patients with poor prognosis.

## 1 INTRODUCTION

Autonomic impairment is a hallmark of heart failure (HF), regardless of the HF phenotype [1-4]. This imbalance is thought to be reflected in increased sympathetic nervous system (SNS) activity and decreased parasympathetic nervous system (PNS) activity and is associated with adverse outcomes [1]. Therefore, pharmacological treatments, such as beta-blockade, that improve autonomic balance are of interest, specifically in the management of HF with reduced ejection fraction (EF) (HFrEF).

Heart Rate Variability (HRV) is commonly used to assess autonomic function and is the degree of variation in the length of the inter-beat intervals [5]. Decreased HRV suggests dysregulation of cardiac autonomic control [6] and predicts mortality in patients with HF [7, 8]. HRV can be assessed using time-domain and frequency-domain from spectral analysis [5] and nonlinear methods [9], which provide parameters that reflect the actions of the PNS and SNS at the sinus node [10].

Although there have been studies on general values for each index of HRV analysis in HF patients, there are no systematic reviews that have comprehensively examined representative values for HF phenotypes classified based on left ventricular (LV) EF (LVEF). This systematic review aims to evaluate the utility of HRV metrics based on the hypothesis that increased sympathetic nervous tone and parasympathetic nervous withdrawal are observed in patients with HFrEF.

## 2 MATERIALS AND METHODS

A systematic review of the studies assessing the utility of HRV metrics in HFrEF was conducted. We searched PubMed, Cochrane, and Embase databases with the following combination of terms: “heart failure” AND “heart rate variability” AND each of “SDNN (standard deviation of normal-normal intervals)”, “SDANN (standard deviation of the average normal-normal R-R period)”, “rMSSD (root mean square of successive differences)”, “pNN50 (percentage of RR adjacent intervals differing from each other more than 50 msec)”, “total power (TP)”, “very low-frequency (VLF)”, “low-frequency (LF)”, “high-frequency (HF)”, “low- to the high-frequency ratio (LF/HF)”, or “nonlinear”. We included all studies that reported the metrics of HRV in HFrEF (LVEF<40%) or HF with LVEF <45%. All eligible studies were retrieved in full forms and analyzed independently by investigators for appropriate inclusion in the systematic review. The research protocol was prepared on the basis of the 2020 PRISMA guidelines and was not registered.

Time domain measures are the simplest to calculate and include the mean normal-to-normal intervals during the entire recording. Since they are always calculated the same way, data collected by different researchers are comparable [11, 12]. The SDNN is the standard deviation of the normal sinus-initiated interbeat interval measured in milliseconds. The SADNN for each of the 5-minute segments during a 24-hour recording was measured and reported in milliseconds like the SDNN. The rMSSD was obtained by first calculating each successive time difference between heartbeats in milliseconds. Then, each of the values was squared, and the result was averaged before the square root of the total was obtained. The pNN50 is the percentage of adjacent normal-to-normal intervals that differ from each other by more than 50 ms [11, 12].

In frequency domain measures, analogous to the electroencephalogram, Fast Fourier Transformation can be used to separate HRV into its component VLF, LF, and HF rhythms that operate within different frequency ranges. This is analogous to a prism that refracts light into its component wavelengths [11, 12]. The VLF band is the power in the HRV power spectrum range between 0.0033 and 0.04 Hz. The LF band ranges between 0.04 and 0.15 Hz. This region was previously called the “baroreceptor range” or “mid-frequency band” by many researchers since it primarily reflects baroreceptor activity while at rest. The HF spectrum is the power in the range from 0.15 to 0.4 Hz. This band reflects parasympathetic or vagal activity and is frequently called the respiratory band because it corresponds to the HR variations related to the respiratory cycle. The ratio of LF to HF power was originally based on 24-hour recordings, during which both PNS and SNS activity contribute to LF power, and PNS activity primarily contributes to HF power. Total power is the sum of the energy in the ultra LF, VLF, LF, and HF bands for 24 hours and the VLF, LF, and HF bands for short-term recordings [11, 12].

Non-linearity means that a relationship between variables cannot be plotted as a straight line. Nonlinear measurements index the unpredictability of a time series, which results from the complexity of the mechanisms that regulate HRV. Nonlinear indices correlate with specific frequency- and time-domain measurements when they are generated by the same processes [12]. A Poincaré plot is graphed by plotting every R–R interval against the prior interval, creating a scatter plot. Poincaré plot analysis allows researchers to visually search for patterns buried within a time series. Unlike frequency-domain measurements, Poincaré plot analysis is insensitive to changes in trends in the R-R intervals [12]. The standard deviation (SD) of the distance of each point from the y = x-axis (SD1) specifies the ellipse’s width. The standard deviation of each point from the y = x + average R–R interval (SD2) specifies the ellipse’s length [12].

Each study was carefully analyzed for methodology (cross-sectional, prospective, or interventional), inclusion and exclusion criteria, LVEF information, HRV measurement duration and methods, and descriptions of baseline values of HRV metrics. Once two investigators independently reviewed all studies, the available data were collected and formulated in tabulated forms for analysis. The primary endpoint in our systematic reviews was to investigate the representative values of the mean or median value of baseline HRV metrics in HFrEF. Studies with interventional design were excluded in this systematic review [13-24].

**Fig. (1):**
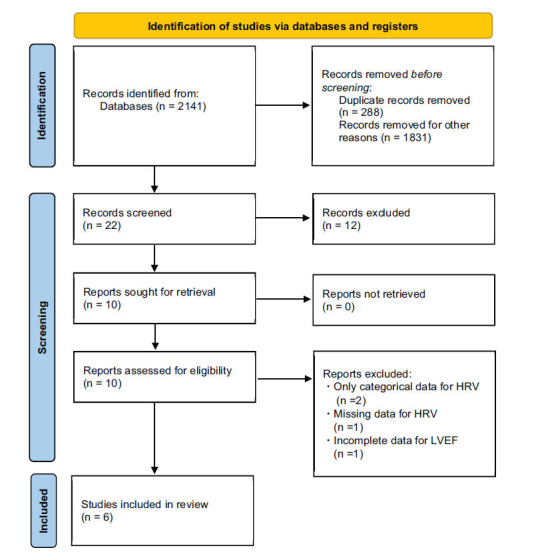
Flow chart of literature search and article selection.

Our search of studies yielded a total of 10 studies (Fig. **1**). Four studies were excluded [25-28]. Among those, only two studies with categorical data for HRV were excluded [25, 26]. One study was excluded due to missing HRV data at baseline [28]. One study with incomplete inclusion criteria for LVEF was excluded [27]. Therefore, the final analysis included six studies with cross-sectional or prospective observational study designs [29-34].

## 3 RESULTS

A total number of 470 patients from 6 studies (1997 to 2010) were finally included in this systematic review. Baseline characteristics are listed in Table **1**. The mean age of the patients was 57.9 years, and most of the studies had a male-dominant population (74 to 88%). The prevalence of ischemic etiology was high, ranging from 57 to 80%. Baseline electrocardiography (ECG) recording time was 24 hours in five studies [29, 31-34] and 2 hours in one study [30]. Time-domain analysis was performed in five studies [29-31, 33, 34], frequency-domain analysis in four studies [29-32], and nonlinear analysis in one study [31].

Table **2** presents the mean or median values for the time-domain analysis. SDNN and SDANN of HFrEF patients were lower in the poor prognosis group [29, 30], the ischemic cardiomyopathy group [31], and the New York Heart Association (NYHA) class II or higher group [34]. Also, the mean or median values of SDNN and SDANN were comparable in the four studies [29, 31, 33, 34] except for the report by Jiang *et al.* [30], in which the ECG recording time was 2 hours. In the other four studies, SDNN and SDANN were derived based on 24-hour ECG recordings [29, 31, 33, 34].

In the study reported by Jiang *et al.* [30], rMSSD was higher in the poor prognosis group compared with the event-free group, whereas in the other four studies, rMSSD was lower in the poor prognosis group [29], ischemic cardiomyopathy group [31], and NYHA class II or higher group [34]. The median rMSSD was the lowest in the study by Jiang *et al.* [30]. pNN50 was lower in the poor prognosis group [29] and ischemic cardiomyopathy group [31], whereas in the study reported by Jiang *et al.* [30], pNN50 was higher in the poor prognosis group compared to the event-free group. On the other hand, among the five studies in which time-domain analysis was performed, the median pNN50 was the lowest in the study reported by Jiang *et al.* [30].

Table **3** presents the mean or median values for the frequency-domain analysis. Except for the median values of LF (ms2) and HF (ms2) calculated from a 2-hour ECG recording period by Jiang *et al.* [30], the mean values of TP, VLF, LF (ms2), LF (log), HF (ms2), HF (log), and LF/HF in HFrEF patients were lower in the poor prognosis group [29], ischemic cardiomyopathy group [31], and LV dysfunction group [32]. In these three studies [29, 31, 32], frequency-domain metrics were derived from 24-hour ECG recordings, and the mean values of LF (log) and HF (log) were comparable. The mean values of the LF/HF ratio were also comparable in the two studies [31, 32].

One study on HFrEF reported a nonlinear index of HRV [31]. SD1 and SD1/SD2 were lower in HFrEF patients with ischemic etiology compared to those with idiopathic etiology, while SD2 was similar between these two groups.

## 4 DISCUSSION

Our systematic review of 6 studies on HFrEF included a total of 470 patients and suggests that time-domain and frequency-domain metrics, calculated from 24-hour Holter recordings, were abnormally lower in patients with HFrEF and were correlated with worse prognosis and advanced symptomatology. Specifically, time domain metrics calculated from 24-hour recordings, such as SDNN, rMSSD, and pNN50, were lower and correlated with worse symptoms and poor prognosis. On the contrary, 2-hour recordings demonstrated a higher rMSSD and pNN50, which correlated with worse outcomes. Another observation was lower time domain metrics in ischemic etiologies for HFrEF compared to non-ischemic causes, although this remains to be further elucidated in future studies. Only two studies described the LF/HF ratio in HFrEF, a metric that has been traditionally linked to the balance of sympathetic and parasympathetic systems. This ratio was found to be abnormally lower in HFrEF compared to normal subjects.

### 4.1 Time-domain Metrics

In the time-domain analysis, SDNN, SDANN, rMSSD, and pNN50 were lower in HFrEF patients with poor prognosis [29], ischemic cardiomyopathy [31], NYHA class II or higher [34], and all of these metrics were calculated from 24-hour Holter ECGs. In one study, rMSSD and pNN50, calculated from 2-hour ECG measurements, were higher in HFrEF patients with poor prognosis [30].

Both SNS and PNS activity contribute to SDNN [35]. Although SDNN is more accurate when calculated over a 24-hour period than over a short period of time, SDNN can be calculated from the standard 5-minute short-time recording [5]. Longer recording periods provide data about cardiac reactions to a greater range of environmental stimuli. Circadian rhythms, core body temperature, metabolism, the sleep cycle, and the renin-angiotensin system contribute to 24-hour HRV recordings, which represent the “gold standard” for clinical HRV assessment [11]. The length of the recording period significantly affects both HRV time-domain and frequency-domain measurements [36]. Since longer recordings are associated with increased HRV, it is inappropriate to compare metrics like SDNN when they are calculated from epochs of different lengths [5]. Generally, resting values obtained from short-term monitoring periods correlate poorly with 24-hour indices, and their physiological meanings may differ [37]. Based on 24-hour monitoring, patients with SDNN values below 50 msec are classified as unhealthy [11]. On the other hand, SDANN is calculated using 5-minute segments during the entire 24-hour time series. Thus, SDANN is not a surrogate for SDNN [11]. Since SDANN does not provide any new information other than SDNN, there might be little need to measure SDANN in addition to SDNN [11]. In this systematic review, most of the studies reported lower SDNN and SDANN in patients with poor prognosis or severe HFrEF [29, 31, 34]. Only one paper reported an SDNN of less than 50 msec, which was derived from 2-hour ECG measurements [30].

The rMSSD is the primary time-domain measurement used to estimate vagally-mediated changes reflected in HRV and is more influenced by the PNS [11]. The conventional minimum recording for rMSSD is 5 minutes, while some researchers have proposed ultra-short recordings of 10 seconds [38]. Furthermore, 24-hour rMSSD measurements are strongly correlated with HF power [12]. Low rMSSD values have been shown to be a risk for sudden unexplained death [39], and less than 33.5msec was associated with poor prognosis [40]. The pNN50 is closely related to PNS activity [35] and is also correlated with rMSSD and HF power. pNN50 less than 14% was found to be associated with poor prognosis [40].

An earlier study reported that HFrEF patients with higher NYHA classes have lower rMSSD values [34]. In the study by Jiang *et al.* [30], although no significant difference was observed in rMSSD and pNN50 between the poor prognosis group and the event-free group, these indexes were lower in the event-free group. NYHA class was higher in the event-free group, suggesting that this may have influenced the results.

In this review, rMSSD and pNN50, which were calculated from 2-hour ECG measurements, were shorter in one study compared to the other studies. The clinical significance of rMSSD and pNN50 may differ between 2-hour and 24-hour measurement times. The length of the recording period significantly affects time-domain metrics. Longer recordings are associated with increased HRV. Since the PNS is activated during the sleep period, rMSSD and pNN50 tend to show high values on a 24-hour Holter ECG [11]. Moreover, these values are also influenced by age and gender [41]. Except for one study [31], in the more severe group, the value of rMSSD was less than 33 msec in the other four studies [29, 30, 33, 34], and that of pNN50 was less than 14% in the other two studies [29, 30]. In summary, the interpretation of the time domain metrics appeared to be dependent on the duration of recordings. Therefore, caution needs to be employed when utilizing such metrics in research studies. Interpretation of data on SDNN, SDANN, rMSSD, and pNN50%, specifically the last two metrics, need to account for the duration of the recording.

In 24-hour ECG recordings of 1,743 subjects 40 to 100 years of age, a linear decline was observed in SDNN [42], and there was a U-shaped pattern of rMSSD and pNN50 with the nadir between 60 and 69 years [42]. In the study by Jiang *et al.* [30], the representative age of participants was 67 years, which was the highest among the studies included in this systematic review, and the SDNN, rMSSD, and pNN50 were the lowest in the study by Jiang *et al.* [30], which is consistent with the notion that age is associated with time-domain metrics. Age also needs to be considered as a factor that influences time-domain metrics.

### 4.2 Frequency-domain Metrics

Most studies included in this systematic review showed that total power, VLF, LF, HF, or LF/HF was low in HFrEF patients with poor prognosis [29], ischemic cardiomyopathy [31], and LV dysfunction [32]. All these metrics were calculated from 24-hour Holter ECG. In one study, HF calculated from 2-hour ECG measurements was higher in HFrEF patients with poor prognosis [30].

Previous studies reported that PNS activity contributed to VLF output because parasympathetic blockade almost completely abolished VLF. A total of 43 VLF rhythms appeared to be generated by stimulation of intracardiac afferent sensory neurons, and the intrinsic cardiac nervous system contributed to the VLF [11, 43-46]. Although the VLF band requires at least 5 minutes of ECG recording time, it is considered optimal to calculate VLF from 24 hours or more of ECG recordings. Low VLF has been shown to be associated with arrhythmia-related mortality [47].

The LF band primarily reflects baroreceptor activity at rest [48]. LF is produced by both the PNS and SNS [5, 12, 49, 50], primarily by the PNS [51] or by baroreflex activity alone [52]. In resting conditions, LF reflects baroreflex activity and not cardiac SNS innervation [11, 12]. During periods of slow respiration rates, vagal activity easily generates oscillations in the heart rhythms that cross over into the LF range [53, 54]. The LF band can usually be calculated from a 2-minute ECG recording at rest [11]. On the other hand, in ambulatory 24-hour HRV recordings, the LF band reflects SNS activity. Physical activity and emotional stress responses over a 24-hour period cause heart rhythm oscillations that cross over into the lower LF band. In long-term ambulatory ECG recordings, the LF band approximates SNS activity as the SNS activity increases [11].

The HF band reflects vagal activity. Heart rate (HR) accelerates during inspiration and slows during expiration. During inspiration, the central autonomic network inhibits vagal outflow, resulting in speeding the HR. Conversely, during expiration, it restores vagal outflow, resulting in slowing the HR *via* the release of acetylcholine [12]. Total vagal blockade eliminates HF oscillations and reduces power in the LF range [11]. HF power is highly correlated with the pNN50 and rMSSD [55]. The HF is conventionally recorded over a minimum 1-minute resting period. In 24-hour ECG, HF band power increases at night and decreases during the day [48]. Lower HF power is correlated with stress and anxiety. Under controlled resting conditions while breathing at normal rates, log-transformed HF is able to estimate vagal tone [56].

In 24-hour ambulatory HRV recordings, it has been suggested that the LF/HF ratio reflects SNS activity, and that ratio has been controversially reported as an assessment of the balance between SNS and PNS activity [12]. Many researchers have convincingly argued that in the resting state, the LF band reflects baroreflex activity and not the sympathetic innervation of the heart [54, 57-60]. On the other hand, while some orthostatic challenges can produce increased SNS activation and vagal withdrawal, psychological stressors can also result in independent changes in SNS or PNS activity [11]. Therefore, it should be recognized that the clinical significance of LF/HF during short-term resting conditions and LF/HF during 24-hour ambulatory ECG measurements are completely different.

In this systematic review, most of the results showed lower values for LF and LF/HF in patients with poor prognosis or severe HFrEF [29, 31, 32]. Previous reports have pointed out that these indicators may be involved in SNS activity, but recent literature suggests that they do not always reflect SNS activity [11]. In the model of circulatory variability to HF patients with varying baroreflex sensitivity, it has been shown that the main differences in spectral power for both LF and HF between and within subjects are caused by changes in the arterial baroreflex gain, particularly for vagal control of R-R interval and LV stroke output [61]. Therefore, both LF and HF are greatly influenced by the gain of the baroreflex and LV function [61]. Thus, in HFrEF, LF and LF/HF are suggested to be paradoxically reduced despite increased SNS drive because baroreflex sensitivity gain is markedly reduced in HFrEF [61].

### 4.3 Nonlinear Metrics

In this systematic review, only one study on HFrEF reported a nonlinear index of HRV. SD1 and SD1/SD2 were lower in HFrEF patients with ischemic etiology compared to those with idiopathic etiology, while SD2 was similar between these two groups [31].

A Poincaré plot is graphed by plotting every R–R interval against the prior interval. Unlike frequency-domain measurements, Poincaré plot analysis is not sensitive to changes in the trend of the R-R interval [62]. Initially, the Poincaré-plot analysis was a qualitative method [63], but the measurement of the major and minor diameters of the ellipse turned this method into a quantitative one. The transversal axis of SD1 reflects short-time changes in the R-R interval and is directly linked to parasympathetic activity. However, the longitudinal axis of SD2 is not so well defined but seems to be inversely proportional to sympathetic activity. Although some studies [64, 65] reported SD2 alterations when atropine of the parasympathetic block or moxonidine of the sympathetic block was administered, most studies during exercise show a clear relationship between a decrease in SD2 and sympathetic stimulation [66].

The SD2 to SD1 ratio is normally used to assess the interaction between parasympathetic and sympathetic activity [12]. However, its interpretation is unclear because both terms of the ratio increase or decrease simultaneously [12, 67]. Regarding nonlinear indicators, such as SD1 and SD2, there are few reports on HFrEF [68], and future verification seems necessary.

## 5 LIMITATIONS

As the definition of HFrEF varies between studies, results should be interpreted with caution. The studies included in this review were published from 1997 to 2010, and the subjects of the studies included in this review were treated differently from the current guideline treatments for HFrEF [69], so it is unclear whether the interpretation of these results applies to current HFrEF patients. In the study by Jiang *et al.* [30], the number of cases was 26, which is smaller than in other studies, and the possibility of statistical error cannot be denied. Time-domain measures of HRV decline with age [42]. Angiotensin-converting-enzyme inhibitors, angiotensin II receptor blockers, and β-blockers, all known to increase survival in HF, have been shown to improve HRV parameters, such as SDNN, rMSSD, and pNN50 in patients with HF [70-76]. Thus, HF medications, including beta-blockades, might affect the values of HRV metrics. However, such precise information was not provided in half of the studies [30-32] included in this systematic review, making it difficult to compare and discuss the effects of HF medications on HRV metrics across studies. In a meta-analysis of 25 studies involving 1,356 individuals with type 2 diabetes mellitus (T2DM) and 1,576 healthy controls, T2DM patients had significantly lower SDNN, rMSSD, pNN50, LF, and HF. Blood glucose and HbA1c levels, as well as time since T2DM diagnosis, were associated with HRV parameters [77]. Therefore, T2DM and its associated information might affect the values of HRV metrics. However, information on the presence and duration of DM was not provided in four studies [29-32], and information on blood glucose and HbA1c levels was provided in none of the studies included in this systematic review [29-34], making it difficult to compare and discuss the effects of DM on HRV metrics across studies.

## CONCLUSION

In this systematic review, including 6 studies on 470 patients, we noted prognostically valuable information obtained from HRV time and frequency domain metrics in patients with HFrEF. Most of the time-domain and frequency-domain metrics were calculated from 24-hour Holter recordings, and those metrics were lower in patients with poor prognosis or severe HFrEF patients. We also noted that time (rMSSD and pNN50) and frequency-domain (LF/HF ratio) metrics vary depending on the duration of recordings and may need to be interpreted with caution in future studies and account for the time duration of recording. The precise information on sympatho-vagal balance in HFrEF using additional HRV metrics, including nonlinear metrics, might require further investigation, as data appears to be sparse. In this systematic review, most HRV metrics were found to be lower in patients with HFrEF.

## Figures and Tables

**Table 1:** Studies included in the review of heart failure with reduced ejection fraction and heart rate variability.

Author (Yr)	Population (HF Type, Approx. Age Yrs)	Male (%)	Number of HFrEF Patients (n)	Ischemic Etiology (%)	Method of Recording (Duration, Software)	Heart Rate Variability Indices
Ponikowski *et al.* (1997) [29]	LVEF<45%, 58 yrs	88	102	76%	24 hr, Marquette 8500	SDNN, SDANN, rMSSD, pNN50, log LF, log HF
Jiang *et al.* (1997) [30]	HFrEF, 67 yrs	81	26	85%	2 hr, Marquette 8000	SDNN, SDANN, rMSSD, pNN50, LF, HF
Bonaduce *et al.* (1999) [31]	HFrEF, 55 yrs	79	97	59%	24 hr, unknown	SDNN, SDANN, rMSSD, Total power, VLF, LF, HF, LF/HF, SD1, SD2, SD1/SD2
Soejima *et al.* (2000) [32]	HFrEF, 60 yrs, (52 controls, 62 yrs)	74	90	57%	24 hr, Marquette 8000	LF, HF, LF/HF
Musialik-Łydka *et al.* (2003) [33]	HFrEF, 54 yrs, (30 age- and gender-matched controls)	84	105	73%	24 hr, unknown	SDNN, SDANN, rMSSD
Kocaman *et al.* (2010) [34]	HFrEF, 63 yrs	74	50	74%	24 hr, unknown	SDNN, SDANN, rMSSD

**Abbreviations:** HF, heart failure; HFrEF, heart failure with reduced ejection fraction; LVEF, left ventricular ejection fraction; SDNN, standard deviation of NN intervals; SDANN, standard deviation of the normal-normal R-R period; rMSSD, root mean square successive difference; pNN50, percent of difference between adjacent normal RR intervals greater than 50 ms; VLF, very low frequency; LF, low frequency; HF, high frequency; SD, standard deviation.

**Table 2:** Baseline resting values of time domain indices of heart rate variability in each clinical study.

-	Ponikowski *et al.* (1997) [29]	Jiang *et al.* (1997) [30]	Bonaduce *et al.* (1999) [31]	Musialik-Łydka *et al.* (2003) [33]	Kocaman *et al.* (2010) [34]
SDNN (msec)	114 ± 38 (Survivor) 84 ± 42 (Cardiac death)	51 [42-75] (Event-free) 40 [26-43] (Had Event)	116 ± 47 (Idiopathic) 111 ± 35 (Ischemic)	147 ± 18 (Control) 85 ± 30 (HFrEF patients)	121 [54-211] (NYHAI) 61 [10-234) (NYHA ≥II)
SDANN (msec)	107 ± 39 (Survivor) 74 ± 38 (Cardiac death)	43 [30-62] (Event-free) 30 [15-35] (Had Event)	101 ± 51 (Idiopathic) 93 ± 31 (Ischemic)	137 ± 21 (Control) 75 ± 26 (HFrEF patients)	83 [32-185] (NYHAI) 51 [4-258] (NYHA ≥II)
rMSSD (msec)	21 ± 11 (Survivor) 19 ± 9 (Cardiac death)	16 [11–28] (Event-free) 19 [14–25] (Had Event)	62± 39 (Idiopathic) 46±26 (Ischemic)	30 ± 6.3 (Control) 26 ± 13 (HFrEF patients)	32 [9-214] (NYHAI) 19 [6-204] (NYHA ≥II)
pNN50 (%)	3.4 ± 4.9 (Survivor) 3.0 ± 3.8 (Cardiac death)	1.3 [0.4–7] (Event-free) 1.9 [1.1–4.8] (Had Event)	14±13 (Idiopathic) 11±10 (Ischemic)	-	-

**Abbreviations:** SDNN, standard deviation of NN intervals; SDANN, standard deviation of the normal-normal R-R period; rMSSD, root mean square successive difference; pNN50, percent of difference between adjacent normal RR intervals greater than 50 ms; HFrEF, heart failure with reduced ejection fraction; NYHA, New York Heart Association.

**Table 3:** Baseline resting values of frequency domain indices of heart rate variability in each clinical study.

-	**Ponikowski *et al.* (1997) [** **29** **]**	**Jiang *et al.* (1997) [** **30** **]**	**Bonaduce *et al.* (1999) [** **31** **]**	**Soejima *et al.* (2000) [** **32** **]**
Total power (ms^2^)	-	-	7044 ± 3626 (Idiopathic) 6168 ± 1947 (Ischemic)	-
Very low-frequency (ms^2^)	-	-	1597 ± 1047 (Idiopathic) 1376 ± 489 (Ischemic)	-
Low-frequency (ms^2^)	-	4.2 [3.5–4.8] (Event-free) 4.2 [3.4–5.1] (Had Event)	620 ± 440 (Idiopathic) 482 ± 206 (Ischemic)	-
(log)	5.0 ± 1.0 (Survivor) 4.1 ± 1.7 (Cardiac death)	-	6.22 ± 0.66 (Idiopathic) 6.06 ± 0.53 (Ischemic)	5.98 ± 0.74 (Normal) 4.56 ± 0.53 (LV dysfunction)
High-frequency (ms^2^)	-	3.4 [2.7–4.1] (Event-free) 3.6 [3.1–4.3] (Had Event)	394 ± 237 (Idiopathic) 300 ± 157 (Ischemic)	-
(log)	3.9 ± 0.9 (Survivor) 3.5 ± 1.1 (Cardiac death)	-	5.76 ± 0.63 (Idiopathic) 5.53 ± 0.56 (Ischemic)	4.98 ± 0.92 (Normal) 3.90 ± 1.03 (LV dysfunction)
LF/HF	-	-	1.59 ± 0.45 (Idiopathic) 1.73 ± 0.56 (Ischemic)	3.21 ± 2.2 (Normal) 1.00 ± 0.27 to 2.68 ± 1.32 (LV dysfunction)

**Abbreviations:** LF, low frequency; HF, high frequency; LV, left ventricular.

## Data Availability

The authors confirm that the data supporting the findings of this research are available within the article.

## LIST OF ABBREVIATIONS

HF: Heart Failure
HRV: Heart Rate Variability
PNS: Parasympathetic Nervous System
SNS: Sympathetic Nervous System

## STANDARDS OF REPORTING

PRISMA methodology and guidelines were followed.

## SUPPLEMENTARY MATERIAL

PRISMA checklist is available as supplementary material on the publisher’s website along with the published article.
